# Canmei Formula Reduces Colitis-Associated Colorectal Carcinogenesis in Mice by Modulating the Composition of Gut Microbiota

**DOI:** 10.3389/fonc.2019.01149

**Published:** 2019-11-19

**Authors:** Huayue Zhang, Dengcheng Hui, Yuan Li, Guangsu Xiong, Xiaoling Fu

**Affiliations:** ^1^Department of Medical Oncology, Yueyang Hospital of Integrated Traditional Chinese and Western Medicine, Shanghai University of Traditional Chinese Medicine, Shanghai, China; ^2^Department of Cirrhosis, Shuguang Hospital, Shanghai University of Traditional Chinese Medicine, Shanghai, China; ^3^Endoscopic Center, Yueyang Hospital of Integrated Traditional Chinese and Western Medicine, Shanghai University of Traditional Chinese Medicine, Shanghai, China

**Keywords:** Canmei formula (CMF), traditional Chinese medicine, gut microbiota, AOM/DSS, colorectal carcinogenesis

## Abstract

The gut microbiota, including pathogenic microorganisms and probiotics, has been involved in tumor initiation and progression by regulating the components of intestinal flora. Canmei formula (CMF), a traditional Chinese medicine, chronicled in the Chuang Yang Jing Yan Quan Shu, has been clinically used as an adjuvant therapy to treat patients with colorectal carcinoma (CRC) in China. In this study, we investigate the treatment effect of CMF in the azoxymethane (AOM) and dextran sodium sulfate (DSS) induced and high-fat diet augmented colitis-associated colorectal cancer *in vivo*, and explore its mechanism of action. We found that CMF treatment relieved the inflammation and alteration of the gut microbiota and significantly inhibited the development of intestinal adenoma. Linear discriminant analysis showed that the flora diversity in the normal mice, model mice and CMF treatment mice was different. At the family level, the relative abundance of Desulfovibrionaceae decreased in CMF groups. The relative abundance of Desulfovibrionaceae were lower in the CMF groups than in model group, whereas Rikenellaceae and Alistipes were increased. Altogether our results indicate that CMF treatment ameliorate colitis-associated colorectal carcinogenesis by modulating the composition of the gut microbiota *in vivo*.

## Introduction

Colorectal carcinoma (CRC) is the second leading cause of cancer-related death worldwide (1). There are about 1.4 million new CRC patients and 700,000 deaths each year (2). Colorectal adenoma (CRA) refers to any lesion that originates from the surface of colorectal mucosa and protrudes into the intestinal cavity. It is a precancerous lesion of CRC and has the characteristics of high recurrence and high incidence of cancerous change (3). Microscopic removal is currently the main treatment for CRA, which can effectively reduce the incidence of CRC and reduce mortality by 67% (4). However, microscopic resection did not reduce the recurrence rate of CRA. Aspirin, celecoxib, and cyclooxygenase-2 inhibitors are used clinically to treat inflammatory bowel disease, but the efficacy is limited according to the large sample clinical data (5). To date, there is no therapeutic agent specific for this disease. It is critical to explore drugs that can safely and effectively prevent and treat the recurrence of CRA.

In China, traditional Chinese medicine (TCM) has a long history dating back several thousands of years ago. Results from both pre-clinical laboratory and human studies have indicated that TCM has been widely used in anticancer therapy. Previous studies have indicated that PHY906, a modified formulation derived from Huang-Qin-Tang, could ameliorate chemotherapy-induced GI tract toxicity (6, 7). Qingjie Fuzheng granules (QFGs) has been proven to be able to inhibit proliferation and induce apoptosis of CRC cells (8). These studies indicate that TCM play important roles in cancer treatment and could be expected to become a promising therapeutic candidate for tumor.

Over the past decade, CRC have emerged as one of the most studied human conditions link to gut microbiota. Studies indicate that changes in the composition of gut microbiota, one of the most important internal environmental factors, are closely associated with the progression of CRC (9, 10). At present, the study on the secretion of toxin-activated inflammation by the intestinal flora is the most in-depth study of CRA carcinogenesis (3). The changes of intestinal flora are positively correlated with colorectal cancer. The secretion of toxins by the bacteria is a key factor for activating inflammation and oxidative stress pathways (11, 12). A number of studies have confirmed that *Enterococcus faecalis, Escherichia coli*, Enterotoxin-producing *Bacteroides fragilis* (ETBF), *Streptococcus bovis, Fusobacterium nucleatum*, and *Helicobacter pylori* are the main species that can induce cancer (3, 12, 13). Among them, *Fusarium nucleatum* induces uncontrolled intestinal epithelial cell growth by activating β-catenin; *E. coli* produces colibactin with genotoxicity (3).

Canmei formula (CMF), a classical traditional Chinese herbal formulation with Titanium and Marci Hieronymi, is widely used to treat colorectal diseases. The active constituents in CMF, including Citric acid, DL-TYROSINE, L(-)-Carnitine, L-Tyrosine, Ambrosic acid and others, have been reported (14, 15). However, there is no study on the effect of CMF in modulating gut microbiota. In our previous studies, 16sDNA sequencing technique was used to compare the change of gut microbiota induced by CMF treatment in AOM/DSS and high-fat diet-induced CRC mice (16, 17). This study provides evidence of the modulations of gut microbiota through CMF treatment, which will help understanding the host-microbe interactions during the treatment of CRC.

## Materials and Methods

### Preparation of the Extracts for CMF

Mume Sieb and Marci Hieronymi were purchased from Yueyang Hospital of Integrated Traditional Chinese and Western Medicine, Shanghai University of Traditional Chinese Medicine. Mume Sieb was the fruit of *Prunus mume*. The Marci Hieronymi is a dry body of the larvae of the camphor family, *Bombyx mori* linnaeus, 4 ~ 5 years old, infected with *Beauveria bassiana*. They were formally identified by the department of pharmacy, Yueyang Hospital of Integrated Traditional Chinese and Western Medicine, Shanghai University of Traditional Chinese Medicine, Shanghai, China.

CMF was formulated with Titanium and Marci Hieronymi in a ratio of 1:1. All the herbs were purchased from Yueyang hospital herbal pharmacy. Formulation was performed as described below. Briefly, 980 g mixture was extracted twice for 1.5 and 1 h for each time. The filtrates were concentrated in a vacuum at 60°C and dried to obtain the CMF extract powder of 335.2 g, the rate of the extract was 32.1%.The extract obtained in this part is CMF-L/H. The same process was repeated twice for the CMF extract. Then the liquid was poured through the column of the macroporous resin, and then the resin column was rinsed with pure water, 20% ethanol and 90% ethanol. After alcohol precipitation, the filtrateswere concentrated in a vacuum at 60°C and dried to obtain the CMF extract powder of 60.3 g, the rate of the purified product was 18%. The extract obtained in this part is used as CMF-A. The quality of preparations was controlled according to the guidelines from Chinese food and drug administration (CFDA).

### Liquid Chromatograph and Mass Spectrometry

The solution obtained by the above preparation method was stored at 4°C. For HPLC analysis, the solution was centrifuged at 12,000 rpm for 10 min, then the supernatant was filtered through a 0.22 μm membrane before injection into the HPLC system. Analysis were performed by using Dionex UltiMate 3000 HPLC system (ThermoFisherScientific, USA) with a diode array detector. The C18 column (150 × 2.1 mm, 2.5 μm) was used with a flow rate of 0.3 ml·min^−1^. The injection volume was 5 μl, and the column temperature was maintained at 40°C. The mobile phase was composed of (A) aqueous formic acid (0.1%, v/v) and (B) acetonitrile (0.1%, v/v) under following gradient elution: 95% A from 0 to 0.5 min, 95-2% A from 0.5 to 15 min, 2% A from 15 to 17 min, 2-95% A from 17 to 17.5 min, 95% A from 17.5 to 25 min. Mass spectrometry was performed on a Q Exactive high-resolution benchtop quadrupole Orbitrap mass spectrometer (Thermo Fisher Scientific, USA) using a heated electrospray ionization (HESI) source for ionization of the target compounds in positive and negative ion modes. The key parameters were as follows: ionization voltage, +3.5 kV/−3.2 Kv; sheath gas pressure, 40 arbitrary units; auxiliary gas, 10 arbitrary units; heat temperature, 350°C. For the compounds of interest, a scan range of m/z 120–1,500 was chosen. Resolution for higher energy collisional dissociation cell (HCD) spectra was set to 17,500 at m/z 120 on the QExactive.

### Animal Experiments and Grouping

The animal procedures and care in this study was approved by the IRB of Yueyang Hospital of Integrated Traditional Chinese and Western Medicine, Shanghai University of Traditional Chinese Medicine. Seven-week-old female C57/BL6 mice were purchased from Shanghai Jihui Experimental Animal Breeding Co., Ltd. After arrival, the animals were quarantined for the first 7 days. Under controlled conditions: humidity 50 + 10%, 12/12 h light/dark cycle, and temperature ~23°C, all animals were housed in plastic cages (6 mice/cage) and provided with free drinking water and basic diet. AOM, the colonic carcinogen, was purchased from Sigma-Aldrich, USA. DSS with a molecular weight of 36,000–50,000 (No.216011080) was purchased from MPBIO Company, USA. For the induction of tumorigenesis, mice were injected with AOM (12 mg/kg body weight) intraperitoneally. Starting 5 day after the injection, animals received 2.5% DSS in the drinking water for 5 days. At the same time, the mice were given a high-fat diet. Then mice were given normal drinking water and normal diet for 2 weeks and subjected to two more DSS treatment cycles. Subsequently, mice were randomized into experimental and control groups. Group 1, NC, *n* = 8. Experimental groups included 5 groups: group 2: treatment with AOM and DSS (MC, *n* = 8); group 3: treatment with AOM, DSS, and Aspirin (Aspirin, *n* = 8); group 4: treatment with AOM, DSS, and CMF ethanol extract (CMF-A, *n* = 8); group 5: treatment with AOM, DSS, and CMF aqueous extract (CMF-L, *n* = 8); group 6: treatment with AOM, DSS, and CMF water extract high dose group(CMF-H, *n* = 8). Aspirin was given every day at the dose of 1.4 mg/kg/d. CMF-L and CMF-H were given at the doses of 3.65 and 7.3 g/kg/d, respectively. CMF-A was given at the doses of 0.657 g/kg/d. In the clinical practice of Chinese herbal medicine, the prescription dose of CMF is usually 60 g of herbal materials per day. When this human dose was converted into an animal dose (a person of 60 kg, and a conversion factor of 9.1 between human and mouse), it was equivalent to the low dose (3.65 g/kg/d) used in this study. All animals were sacrificed at the 18th week after the corresponding drug was administrated.

### Colon Tissue Collection and Processing

After mice were sacrificed, the colorectal adenoma tissues were rapidly isolated, freed of adherent tissues and rinsed with phosphate buffer saline. Subsequently, tumor biopsy specimens of 1 cm-length were fixed with 4% paraformaldehyde for 24 h, embedded in paraffin and sectioned into 4-μm-thick slices. Slices were hydrated and stained with hematoxylin and eosin (H&E) followed by microscopic examination. Then portions of adenoma tissues were homogenized (10% W/V) in (20 mM Tris-HCI containing 1 mM EDTA, PH 7.4) by a glass homogenizer. The homogenates were centrifuged at 3,000 × g for 20 min at 4°C and supernatants were stored at −80°C.

### Real Time Quantitative Reverse Transcription PCR

According to the modified methods as described, the RNA extracts of the colorectal tissues were prepared (8). By normalizing target mRNA *Ct* values to those for GAPDH (Ct), the relative amount of target mRNA was determined by the comparative threshold (Ct) method. The primer sequences used were as follows:

MUS-NF-κB-F (bp1297): (ATGCACCGTAACAGCAGGAC);
MUS-NF-κB-R (bp1405C): (TGTCATCCGTGCTTCCAGTG);
MUS-IL-17C-F (bp269) (AGGAGGTGCTGGAAGCTGAC);
MUS-IL-17C-R (bp391C) (TGCATCCACGACACAAGCAT);
GAPDH-F (5′-GTGAGGCCGGTGCTGAGTAT-3′);
GAPDH-R (5′-GTGCAGGATGCATTGCTGAC-3′);

### ELISA Assay

Concentration of IL-17C in serum and NF-κB in supernatants of colonic tissues were quantified by IL-17C assay kit and NF-κB assay kit (Shanghai Pusheng Biological Technology Co., Ltd. China) according to manufacturer's instruction.

### Fecal DNA Extraction and Illumina Miseq Sequencing

Genomic DNA was extracted from every stool sample using the FastDNA Spin Kit for Soil (MP Biomedical, LLC, catalog 116560-200) following the manufacturer's instructions.V3 and V4 region of the 16s rDNA sequencing was amplified by Primer F (5′-AACGGGAAGACAACGTACGG-3′) and Primer R (5′-CAGATGCAGGAGGACATGTC-3′) with barcode sequence. Library was constructed following the manufacturer's instructions of the Ion Plus Fragment Library Kit and sequenced by Ion S5 Sequencer.

### Bioinformatics Analysis

Reads was filtered and chimera sequence was removed.

The read pairs were de-multiplexed based on their unique molecular barcodes and overlapping reads were merged using USEARCH v7.0.1001 software. Operational taxonomic unit (OTU) picking was conducted using the QIIME (Quantitative Insights Into Microbial Ecology) software package. By using UCLUST, 16S rRNA gene sequences were clustered at a similarity cutoff value of 97%. By the SILVA reference database (https://www.arb-silva.de/download/), matching of OTUs to bacteria was conducted. By mapping the de-multiplexed reads to the identified OTUs, abundances were recovered. Alpha andbeta diversity plots were also generated using QIIME. The non-weighted UniFrac approach was used to measure the beta diversity between five bacterial cave communities. The relative abundance of transporter genes was predicted and performed by picrust analysis. Independent of the taxonomic analysis, aclosed-reference OTU picking protocol (QIIME) and the Greengenes database (http://greengenes.lbl.gov) were used to pre-cluster at 97% identity, and 97% of the OTUs were picked. The obtained OTU table was normalized by 16S rRNA copy number, and the functional genes were predicted according to the Kyoto Encyclopedia ofGenes and Genomes (KEGG) catalog. By using SPSS version 15.0 statistical software (IBM, Armonk, NY, USA), Fisher's test and the Mann-Whitney U testwere used to analyze, respectively, the gender distribution of subjects and intergroup differences at the genus level in each subcluster. The linear discriminant analysis (LDA) effectsize (LEfSe) method with default settings was used to analyze the differences among groups in phyla, class, order, family, and genera levels.

### Statistical Analysis

SPSS software version 15.0 was used for statistical analysis. The data were presented as mean ± SD. Unpaired students' *t*-test was used to compare the means of the two groups. One-way analysis of variance and Adonis were used to compare the means of more than two groups. A level of *P* < 0.05 was considered as statistically significant.

## Results

### Identification of the Main Components in the Extract of CMF

**Figure 1:**
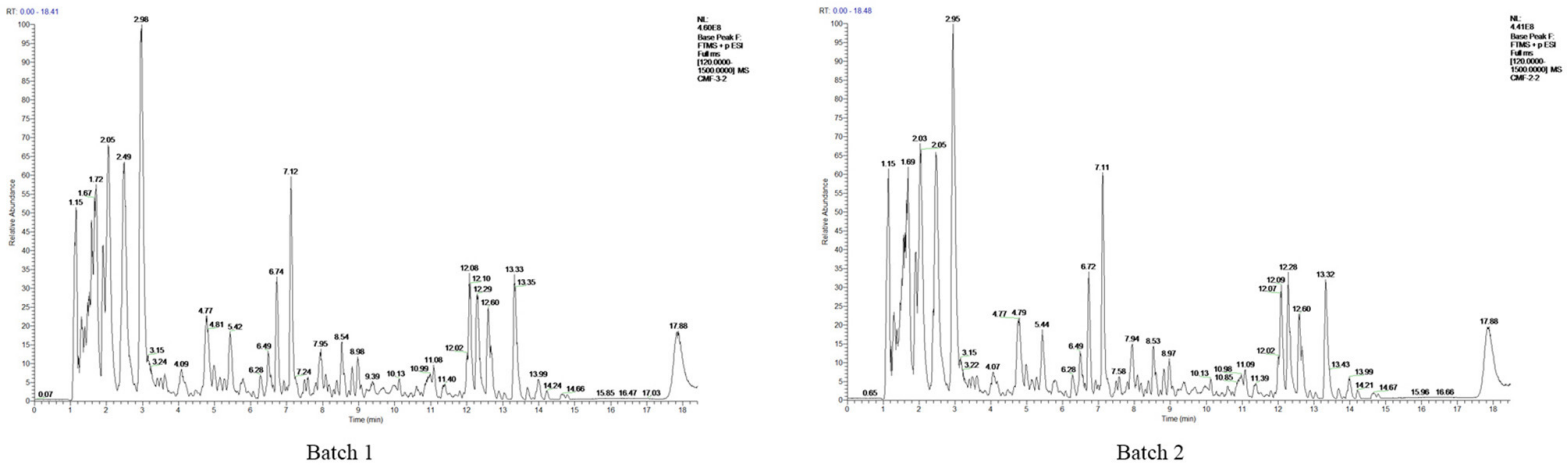
HPLCprofile of two batches of CMF. The chemical fingerprint of CMF batch #1 and batch #2 was measured by HPLC analysis.

To identify the components of Mume Sieb and Marci Hieronymi extract, Q Exactive high-performance benchtop quadrupole-Orbitrap LC-MS/MS was performed. The main components in the extract of CMF were identified according to the elemental composition data determined from accurate mass measurements and comparison with the literature data. Forty-one compounds were identified in the CMF extract and demonstrated in Table 1. In addition, to exclude the possibility of manufacturing problems relating to procession, extraction, handling, and storage, two batches of CMF were evaluated by HPLC. We found that the magnitude, number, and retention time of the peaks were similar between the two batches (Figure 1). These results confirmed the stability of the CMF extraction process.

**Table 1:** Major chemical constituents identified in CMF.

**No**.	**Name**	**RT (min)**	**Formula**	**Molecular weight**	**Area (max.)**
1	DL-Arginine	1.28	C6 H14 N4 O2	174.1117	1300061.86
2	L(-)-Carnitine	1.37	C7 H15 N O3	161.10527	9750518.42
3	DL-Stachydrine	1.40	C7 H13 N O2	143.09471	1508022.19
4	Adenine	1.42	C5 H5 N5	135.05457	12469462.57
5	Guanine	1.43	C5 H5 N5 O	151.04946	7435505.89
6	Hypoxanthine	1.57	C5 H4 N4 O	136.03858	12616671.18
7	Nicotinic acid	1.58	C6 H5 N O2	123.03221	1086991.17
8	L-Tyrosine	1.64	C9 H11 N O3	181.07401	6502680.25
9	Adenosine	1.69	C10 H13 N5 O4	267.09676	26955456.82
10	Adenine	1.69	C5 H5 N5	135.05457	3750819.71
11	2-Hydroxycinnamic acid	1.72	C9 H8 O3	164.04745	37877896.83
12	Hypoxanthine	1.76	C5 H4 N4 O	136.03858	2774356.88
13	L-Norleucine	2.07	C6 H13 N O2	131.09474	55189905.01
14	Alanyltyrosine	2.81	C12 H16 N2 O4	230.12691	433071.84
15	Nornicotine	2.88	C9 H12 N2	148.10023	477190.87
16	2-Oxindole	3.52	C8 H7 N O	151.06347	420066.93
17	2′-Deoxyadenosine	3.54	C10 H13 N5 O3	251.10184	148660.32
18	Glycyl-L-leucine	4.17	C8 H16 N2 O3	188.11627	4092286.76
19	Chlorogenic acid	4.80	C16 H18 O9	354.09487	36388021.14
20	tert-Butyl N-[1-(aminocarbonyl)-3-methylbutyl]carbamate	5.41	C11 H22 N2 O3	230.16338	2831504.35
21	D-(+)-Tryptophan	5.45	C11 H12 N2 O2	204.08996	21112273.16
22	3,4-Dihydroxybenzaldehyde	5.90	C7 H6 O3	138.03188	196915.08
23	Leucylproline	5.94	C11 H20 N2 O3	228.14765	2951420.35
24	7-Hydroxycoumarine	6.51	C9 H6 O3	162.03159	3160911.99
25	6-Methylquinoline	6.93	C10 H9 N	143.07372	1669486.73
26	3-Phenylpropanoic acid	7.18	C9 H10 O2	150.06808	50678.13
27	3-O-feruloyl-D-quinic acid	7.63	C17 H20 O9	368.11073	285397.82
28	4-Coumaric acid	7.90	C9 H8 O3	164.04747	616864.76
29	Rutin	7.92	C27 H30 O16	610.15385	383860.64
30	N-Acetyl-L-leucine	7.95	C8 H15 N O3	173.10535	172744.27
31	Quercetin-3β-D-glucoside	8.13	C21 H20 O12	464.09641	532784.34
32	3,5-Dimethoxybenzoic acid	8.22	C9 H10 O4	182.05817	712514.01
33	Quercetin	8.52	C15 H10 O7	302.0428	150731.57
34	Kaempferol-7-O-glucoside	8.53	C21 H20 O11	448.10099	407020.68
35	(3R,4S)-4,6,8-Trihydroxy-7-methoxy-3-methyl-3,4-dihydro-1H-isochromen-1-one	8.89	C11 H12 O6	222.05297	117315.38
36	(2E)-3-(3,4-Dimethoxyphenyl)acrylic acid	9.07	C11 H12 O4	208.07356	2293865.83
37	Ambrosic acid	10.00	C15 H20 O4	264.13609	678788.38
38	Andrographolide	10.34	C20 H30 O5	350.20662	1261013.91
39	Ipratropium	11.70	C20 H29 N O3	331.21202	224430.08
40	Pinolenic acid	13.35	C18 H30 O2	278.22456	293978.53
41	α-Eleostearic acid	14.23	C18 H30 O2	278.22457	1744840.46

### CMF Attenuated AOM/DSS-Induced Colitis-Associated Tumorigenesis

**Figure 2:**
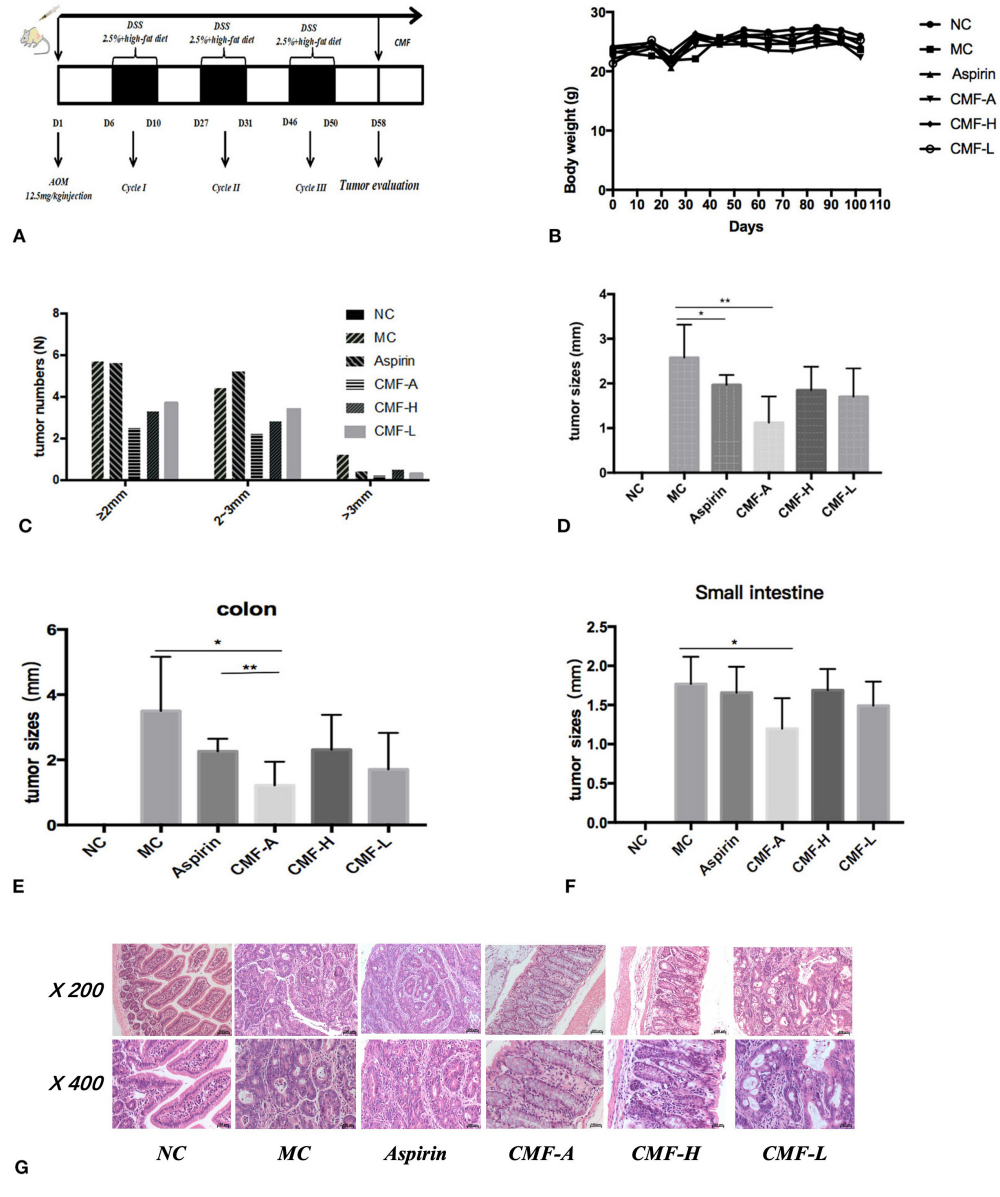
CMF treatment reduced the incidence of CRA in mice. C57BL/6 mice were subjected to an AOM-based CRC induction protocol using three cycles of 2.5% DSS in drinking water. **(A)** Diagram shows the experimental course of AOM/DSS mouse model. **(B)** Body weights of AOM/DSS group and AOM/DSS + CMF group (1, 3, 4, 5, 6). **(C)** Histogram showing the size distribution of tumors. **(D–F)** Tumor sizes in different parts determined by Spot software for microscopic tumors or a caliper for macroscopic tumors. Average tumor size ± S.D. is shown; **(G)** H&E stains of serial sections of colons. *P < 0.05; **P < 0.01. Data are presented as mean ± SD of mice in each group.

To evaluate the protective effect of CMF on the development of colitis-associated cancer, the well-established AOM/DSS and high-fat diet-induced colitis-associated colorectal tumor model in C57BL/6 mice were used (Figure 2A). Initially, the toxicity of CMF was tested *in vivo*. We did not find weight loss or behavioral changes in all mice. These results indicate that each of those extracts of CMF were well-tolerated (Figure 2B). AOM/DSS colorectal cancer mice were induced as described. All mice were treated with CMF once a day. After CMF treatment, tumor development and growth was inhibited in AOM/DSS colorectal cancer mice (Figures 2C–F). As a control, aspirin treatment effectively inhibited the occurrence of intestinal adenomas of 3 mm and above, but had no inhibitory effect on the occurrence of adenomas smaller than 3 mm (Figure 2C). Different extracts of CMF can significantly inhibit the growth of intestinal glands. Figures 2D–F shows that the ethanol extract of CMF significantly inhibited the occurrence of intestinal adenoma, and there is no difference in the occurrence of adenoma in the colon and small intestine. Histological examination revealed that mucosal inflammation, aberrant crypt foci adenoma, ulcer, or dysplasia in the colon tissues of the model group. Histological studies indicated that there was a large adenocarcinoma inside mucosa with several abnormal cells exhibiting cylindrical shape, large nuclei, increasing nuclear/cytoplasmic (N/C) ratio and cellular cleavagein AOM/DSS group (Figure 2G). CMF remarkably relieved the condition. These results suggest that CMF treatment inhibits inflammation-associated carcinogenesis in the AOM/DSS and high-fat diet mice.

### CMF Treatment Repressed NF-κB and IL-17C Signaling in AOM/DSS Induced Mice

**Figure 3:**
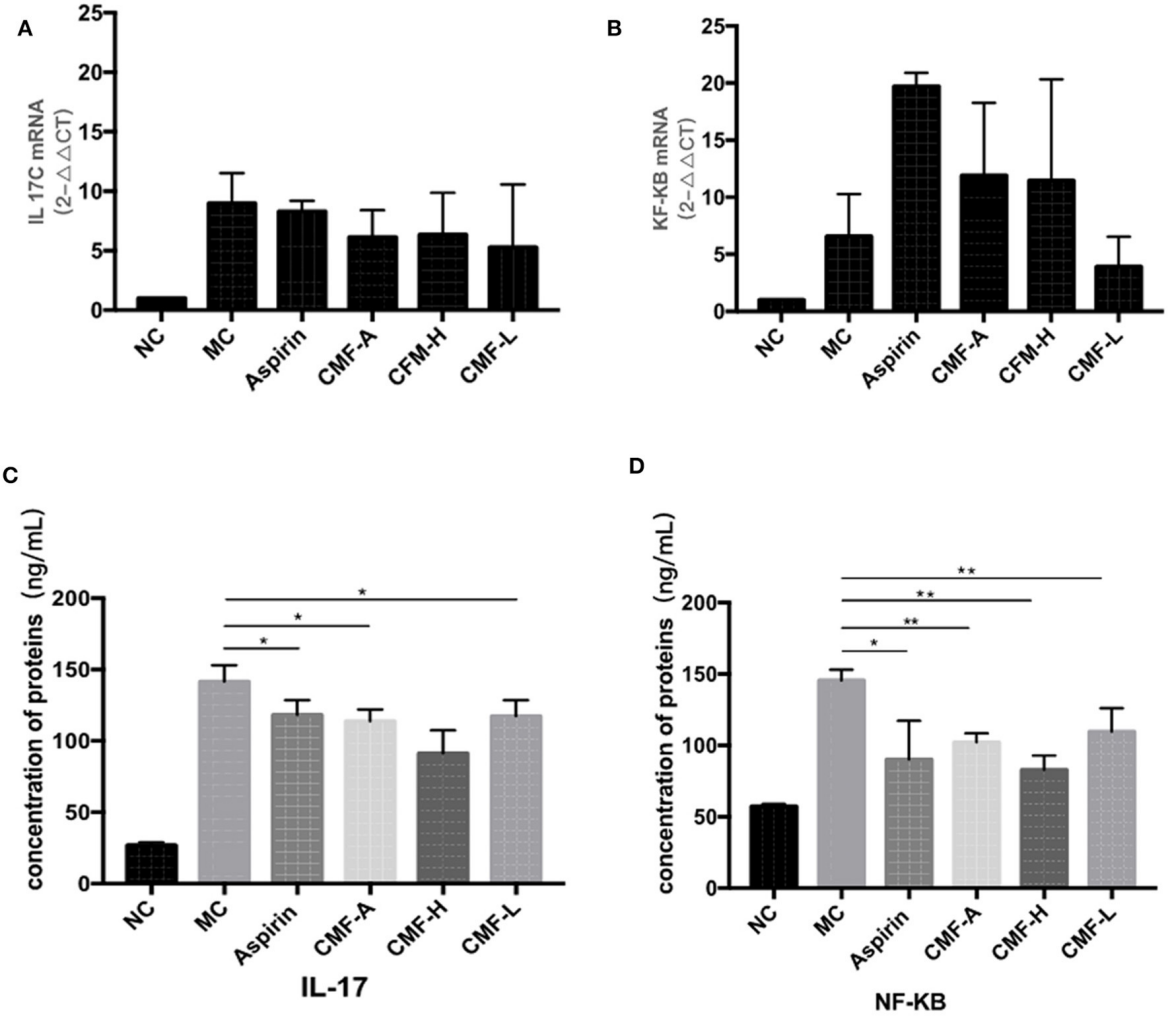
CMF changed the inflammatory microenvironment and regulated the expression of IL-17C and NF-κB *in vivo*. mRNA level of IL-17C **(A)** and NF-κB **(B)** in the colonic tissues were measured by real-time RT-PCR. All data are expressed as the mean ± S.D, *P < 0.05, ^**^P < 0.01 compared with AOM/DSS group. The level of IL-17C in serum **(C)** and the level of NF-κB in tumor tissue homogenate **(D)** were measured using ELISA kits. All data are expression as the mean ± S.D, *P < 0.05, **P < 0.01 compared with AOM/DSS group.

As NF-κB and IL-17C play important roles in the development of CRC, the mRNA expression of IL-17C and NF-κB collected from CRC mice were detected by Quantitative real-time PCR (Figures 3A,B). IL-17C was upregulated in the colorectal tissues of AOM/DSS mice. However, treatment with different extracts of CMF, including water extract and alcohol extract, effectively suppressed the mRNA expression of IL-17C. Drugs treatment, including aspirin, induced the mRNA expression of NF-κB. ELISA assay was performed to detect the protein expressions of inflammatory factors. As it is illustrated in Figure 3C, the protein levels of IL-17C in the serum of AOM/DSS-induced mice were significantly highest among mice with other drug intervention. The level of NF-κB significantly reduced in the tumor tissues of CMF-A, CMF-H, CMF-L, and Aspirin groups when compared to MC group (Figure 3D). All these results indicate that CMF treatment represses the expression of NF-κB and IL-17C.

### CMF Treatment Regulated Gut Microbiota

**Figure 4:**
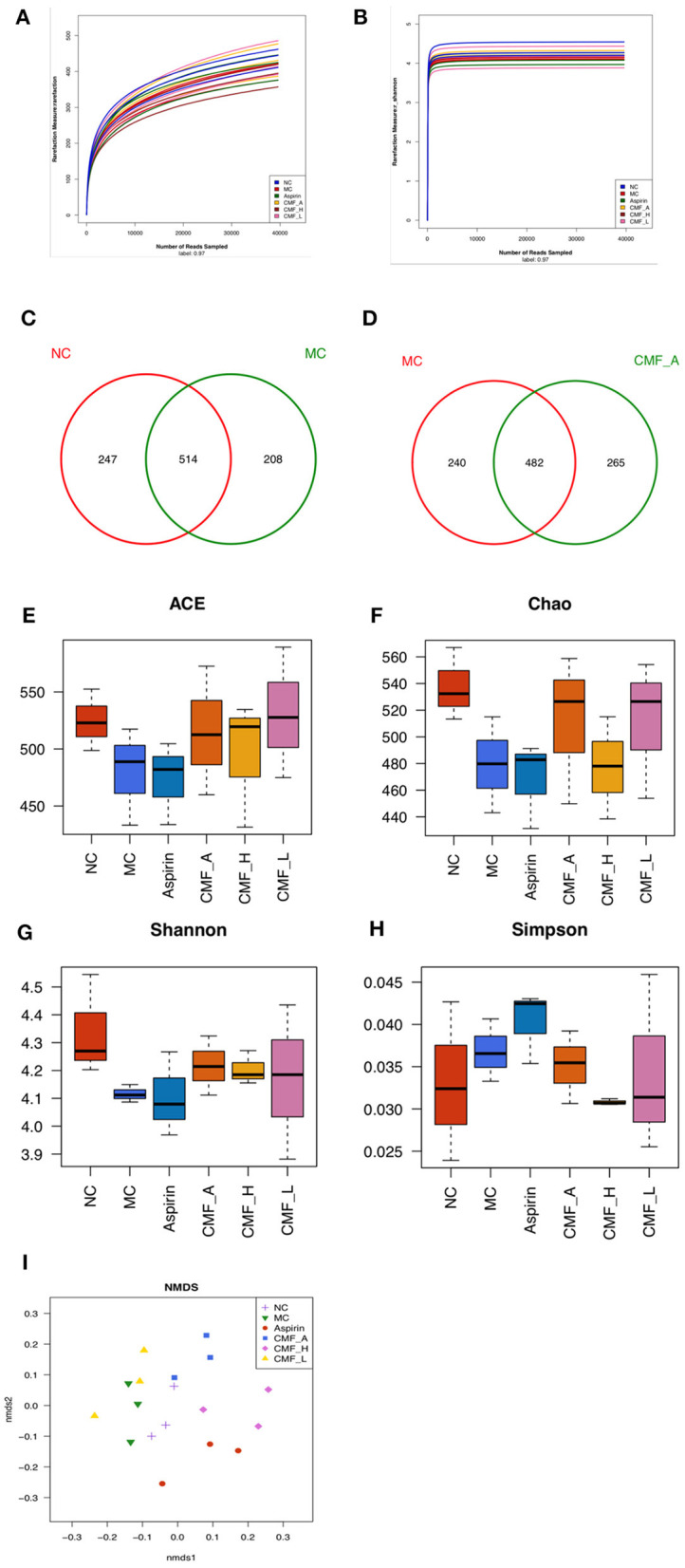
Illumina Miseq sequencing data showed CMF modulates the overall component of gut microbiota. **(A)** Rarefaction curves determined at the 97% similarity level. **(B)** Shannon-Wiener curves of samples, calculated using mothur. **(C–F)** Venn diagram of OUTs in the different groups. **(G–I)** Community richness and diversity index of the model control group (MC), normal control group (NC), aspirin group, and CMF intervention groups (CMF-A/H/L) on ACE, Chao, Shannon, Simpson analysis.

To examine the effect of CMF on the regulation of components of gut microbiota, high-throughput pyrosequencing was performed with an Illumine MiSeq platform to generate 39584 high quality and valid sequences from 16 samples from different groups. We conducted a bar-coded pyrosequencing run to analyze the changes of gut microbiota in the five studied groups. In total, 39,584 available reads and 1461 OUTs were obtained. Shannon diversity and rarefaction curves are shown in Figures 4A,B. The rarefaction curves were consistent with the current sequencing, demonstrating that most of the flora diversity has been captured in all samples. What's more, the overlap of OUTs between groups revealed that 482 OUTs coexisted in both the MC group and CMF-A group, 496 OUTs coexisted in both the NC group and CMF-A group, 514 OUTs coexisted in both the NC group and MC group and 467 OUTs coexisted in both the MC group and Aspirin group (Figures 4C,D). In addition, we examined the flora community richness and diversity in mice. No statistical difference was observed for the ACE, Chao, Shannon, and Simpson indices among these groups (Figures 4E–H), although Simpson index were lower, but other indices are higher in NC group and the different CMF extract groups compared with MC group. Nonmetric multidimensional scaling (NMDS) analysis indicated the changes of overall structure of gut microbiota among different groups (Figure 4I). These results showed that the diversity of bacterial population of the NC group was higher than that of the MC group. There was a statistical difference between the CMF-H group and the MC group. These results suggested that CMF treatment increases the diversity of the intestinal flora.

### Analysis of the Relative Abundance of Microbiota at the Family Level and Genus Level

**Figure 5:**
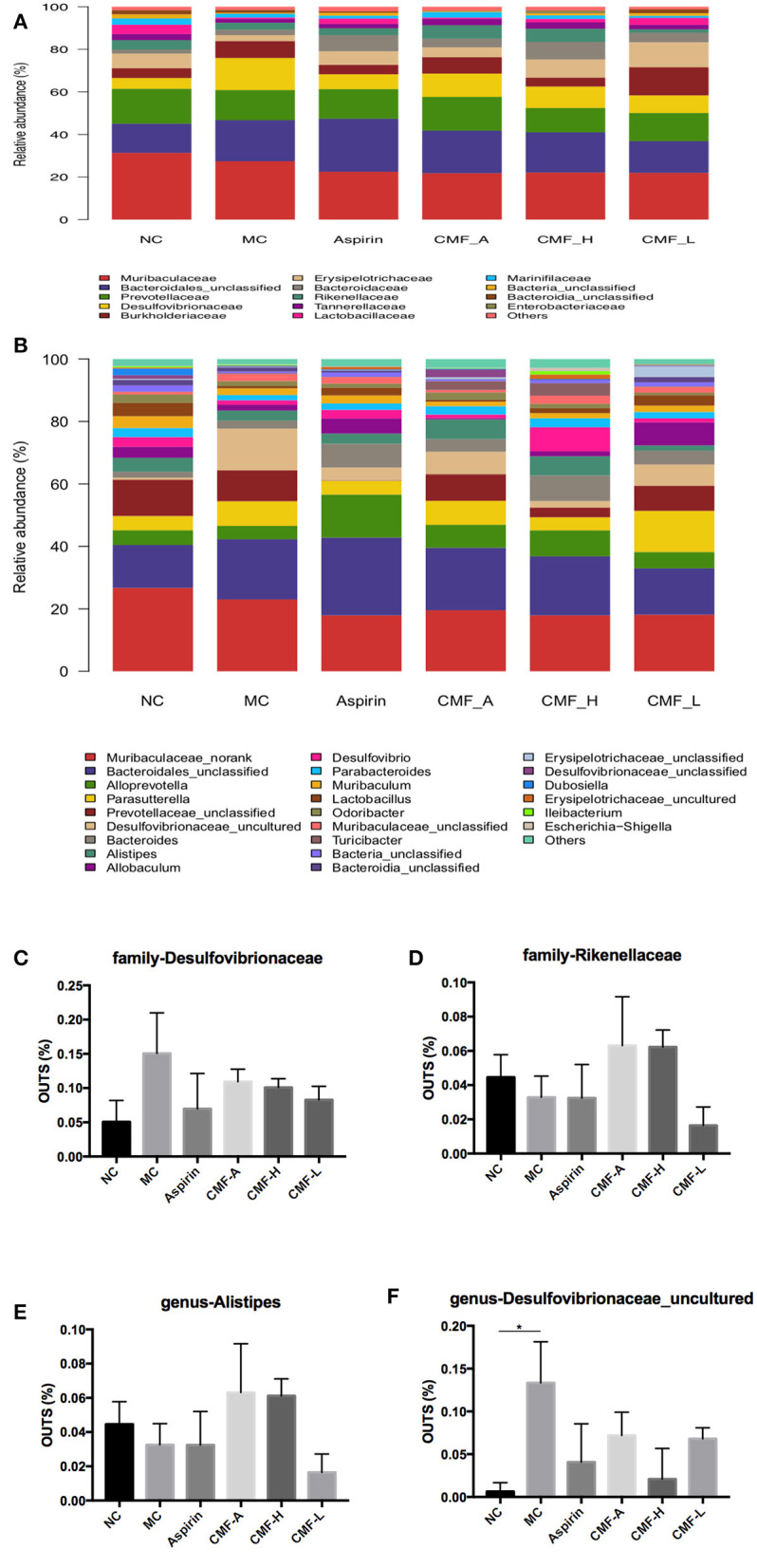
Comparison of gut microbiota among groups. Microbiota distribution at the family level **(A)** and the genus level **(B)**. Relative abundance measured with qPCR for family-Desulfovibrionaceae, family- Rikenellaceae, and genus-Alistipes, genus- Desulfovibrionaceae_uncultured **(C–F)**. Superscript letters indicate statistical differences (*P < 0.05).

The family muribaculaceae, bacteroidales, and prevotellaceae were the most prevalent taxa in these groups (Figure 5A). It was found that these bacteria are dominant bacteria at the genus level (Figure 5B). Intraindividual changes were detected in these groups. Compared with the model group, the abundance of a small number of bacteria increased or decreased to varying degrees, and most of the bacteria remain unchanged. At the family level, abundance of Desulfovibrionaceae decreased in groups that were treated with CMF. Conversely, a transient CMF-treatment increase several family microbiota, including Rikenellaceae, Erysipelotrichaceae, Lactobacillaceae, Streptococcaceae, and Tannerellaceae. At the genus level, the abundance of genus *Alistipes, Faecalibaculum, Lactobacillus*, and *Parabacterioides*, were up-regulated after the treatment of CMF. The genus *Parasutterella* was found down-regulated in CMF treatment groups. It is worth mentioning that genus Desulfovibrionaceae_uncultured acted to AOM/DSS-mediated gut microbiota dysbiosis had statistical difference (Figure 5F). There arechanges in the gut microbiota but there is no statistical difference in these changes (Figures 5C–E).

**Figure 6:**
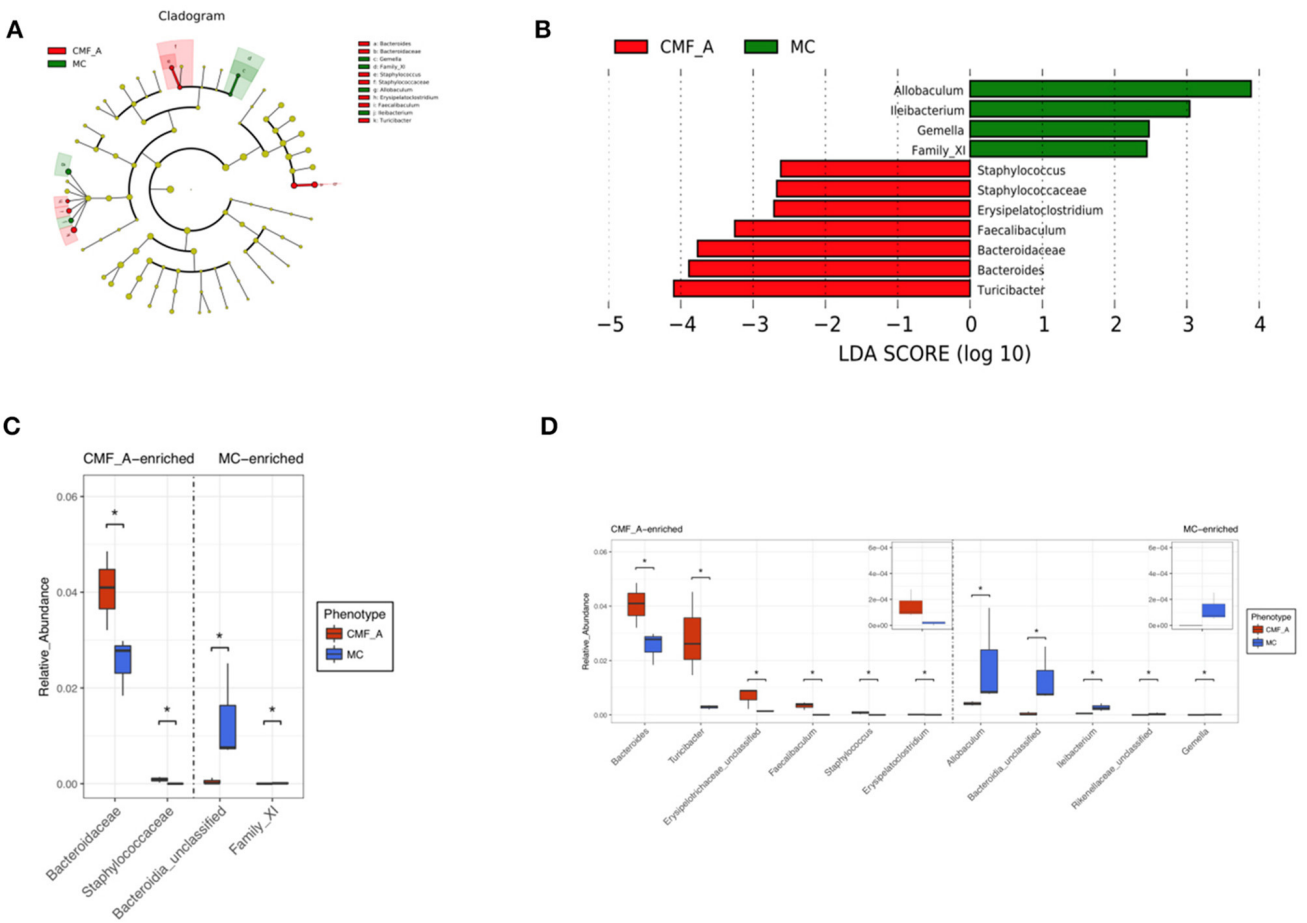
Comparison of gut microbiota between CMF-A group and MC group. Cladogram of linear discriminant analysis (LDA) coupled with effective size measurement showing differentially abundant genera **(A)**. Histogram of LDA scores for differentially abundant genera **(B)**. qPCR identified the difference in CMF-A group and MC group of mice and *t*-test for significance testing at family level **(C)** and genus level **(D)**. Superscript letters indicate statistical differences (*P < 0.05).

To systematically examine the alteration of taxa abundance according to CMF treatment, we conducted LEfSe (LDA Effect Size). The microbial cladogram indicates that the gut microbiome significantly altered in Bacteroidaceae and Staphylococcaceae, ranging from family to genus level (Figures 6A,C). At the genus level, the relative abundance of *Turicibacter, Bacteroides, Bacteroidaceae, Faecalibaculum, Erysipelatoclostridium*, and *Staphylococcus* were enriched in mice treated with the CMF-A diet (Figures 6B,D).

## Discussion

Traditional Chinese medicine (TCM) have been used for the treatment of diseases and health conditions for several thousands of years. In the treatment of CRC, the application of TCM has received worldwide attention. Many studies have demonstrated the anti-tumor effect of TCM in CRC therapy. For example, Wei et al. showed that JianPi JieDu decoction (JPJD) is able to inhibit tumorigenesis, metastasis, as well as angiogenesis of tumors through the mTOR/HIF-1α/VEGF pathway (18). Wu et al. demonstrated that Gegen Qinlian decoction (GGT) can ameliorate gut-toxicity through anti-inflammatory pathways, inhibition of neutrophil migration, anti-oxidative stress via the Nrf2/Keap-1 pathway in the colon (19). Wing lam et al. reported that PHY906 reduced the CPT-11-induced gastrointestinal toxicity by inhibiting multiple steps of inflammation and the promotion of intestinal progenitor cell repopulation (20). Extensive studies have revealed that TCM antitumor drugs work by inhibiting tumor proliferation, promoting tumor apoptosis, and affecting diverse molecular targets (21–23).

The alterations of cancer genetic and molecular targets in CRC treatment have been extensively reported. Recent studies have reported that the regulation of gut microbiota may affect carcinogenesis (24–28). Intestinal flora is a direct factor of intestinal cancer. In this study, we found that CMF can ameliorate colitis-associated colorectal carcinogenesis in mice by modulating the fecal microbiota. The gut microbiota, including *Fusobacterium* (F.) *nucleatum* (29) and Firmicutes, Bacteroidetes Ley et al. (30), plays an important role in human health. In addition, many factors will affect and destroy the balance of intestinal microbiota, including the invasion of antigens, activation of immune cells, and production of cytokines. Therefore, investigating the relationship between CMF and the gut microbiota in AOM/DSS-induced colitis in miceis critical.

In our study, the anti-inflammatory role of CMF in the *in vivo* model of colitis-associated colorectal carcinogenesis induced by AOM/DSS and high-fat diet in C57BL/6 micewere investigated. Two different solvent extraction components of CMF were used to treat these mice to evaluate its therapeutic effect. The results showed that each of these extracts of CMF were well-tolerated. Histopathology findings were consistent with previous experimental results (31). The CRC mice treated with AOM/DSS and high-fat diet presented more severe condition than CMF-treated mice. In contrast, administration of CMF decreased tumor number and tumor size. Especially the ethanol extract of CMF can effectively inhibit the occurrence of colitis-associated colorectal carcinogenesis in the colon and small intestine.

Previous studies showed that tumor-prone mice colonized with *E. coli* and *B. fragilis* and increased IL-17 expression that promotes colon tumorigenesis in AOM mice (3, 32). It was reported that spontaneous activation of NF-κB was detected in tissues of human colorectal cancer (31, 33). We found that CMF could decrease the expression level of IL-17C and inhibit the activity of NF-κB.

High-throughput sequencing determined microbiological composition in mice. Most of the diversity of microorganisms was captured in all samples. The ACE, Chao, Simpson, and Shannon indices reveal that the microbial diversity in control mice and CMF treated mice were greater than in the model mice (16). The microflora analysis show that there are significant differences between the control mice and the model mice. There was a statistical difference between the ethanol extract of CMF treated mice and the model mice. This result indicated that the ethanol extract of CMF could increase the diversity of the intestinal flora. We evaluated phylum, class, order, family, and genus, the gut microbiota community in all samples. From the experiment, Muribaculaceae, Bacteroidales, and Prevotellaceae were the dominant family found in the gut. In accordance with family criteria, these flora occupied a main tier in the genus level. Focus on certain bacteria, statistical results reveal that CMF reversed the gut microbiota dysbiosis in CRC mice, including inhibiting the growth of Desulfovibrionaceae and promoting the Rikenellaceae. Desulfovibrionaceae, a kind of sulfate-reducing and endotoxin-producing bacteria, were mostly enriched in mice with long term high-fat feeding. Previous studies found that the family Desulfovibrionacea is positively associated with obesity (9, 34–36). This phenomenon was also found in our study. Wu et al. found that relative abundances of Rikenellaceae increased in the AOM/DSS mice (32). This result was in consistent with our study. We speculate that Rikenellaceae might be a bacterium that could cause cancer.

Because the results of our study showed that the alcohol extract of CMF is more effective than that of the water extract, we analyzed the difference of flora of the mice treated with the alcohol extract of CMF and the model mice. The results showed that the gut microbiome significantly altered in Bacteroidaceae and Staphylococcaceae, ranging from family to genus level. As shown in our results, at genus level, the relative abundance of *Turicibacter, Bacteroides, Faecalibaculum, Erysipelatoclostridium*, and *Staphylococcus* were enriched in mice treated with the CMF-A diet. In a previous study, the abundance of Bacteroidaceae had an important influence in the active progression of colitis in DSS-treated mice (16), which was in contrast to our study. Liu et al. found that the abundance of *Turicibacter* decreased in obesity mice (37, 38). Similarly, the *Turicibacter* was also reduced in AOM/DSS and high-fat diet mice in this study. Therefore, we suspect that *Turicibacter, Bacteroides, Faecalibaculum, Erysipelatoclostridium*, and *Staphylococcus* were the beneficial bacteria in the gut. But the specific role of these gut microbiota needs to be further investigated.

In summary, we report that CMF, a two-herb formulation, has reduced the incidence of CRC by inhibiting inflammatory factors and regulating gut microbiota. In addition, the mechanism of action of CMF is through the direct action on inflammatory factors or the indirect effect on regulating gut flora. Understanding the role of gut microbiota would be helpful for the prevention of CRC development.

## Data Availability Statement

All datasets generated for this study are included in the article/supplementary material.

## Ethics Statement

The animal study was reviewed and approved by IRB of Yueyang Hospital Affiliated with Shanghai University of Traditional Chinese Medicine. Written informed consent was obtained from the owners for the participation of their animals in this study.

## Author Contributions

XF, HZ, and GX contributed to study design, data interpretation, and manuscript preparation. YL contributed to animal rearing. DH contributed to data acquisition and analysis.

### Conflict of Interest

The authors declare that the research was conducted in the absence of any commercial or financial relationships that could be construed as a potential conflict of interest.
