# Corrigendum: Canmei formula reduces colitis-associated colorectal carcinogenesis in mice by modulating the composition of gut microbiota

**DOI:** 10.3389/fonc.2025.1609683

**Published:** 2025-05-12

**Authors:** Huayue Zhang, Dengcheng Hui, Yuan Li, Guangsu Xiong, Xiaoling Fu

**Affiliations:** ^1^ Department of Medical Oncology, Yueyang Hospital of Integrated Traditional Chinese and Western Medicine, Shanghai University of Traditional Chinese Medicine, Shanghai, China; ^2^ Department of Cirrhosis, Shuguang Hospital, Shanghai University of Traditional Chinese Medicine, Shanghai, China; ^3^ Endoscopic Center, Yueyang Hospital of Integrated Traditional Chinese and Western Medicine, Shanghai University of Traditional Chinese Medicine, Shanghai, China

**Keywords:** Canmei formula (CMF), traditional Chinese medicine, gut microbiota, AOM/DSS, colorectal carcinogenesis

In the published article, there was three errors in “H&E stains of serial sections of colons” of **Figure 2G** as published. There are three errors in the original version of **Figure 2G**, which are as follows:

The first error is that the pathology pictures of the MC group (°C200, blue border in **Figure 2G**-Original Version) and the NC group (°C400, red border in **Figure 2G**-Original Version) are the HE staining results of the CMF-A group (°C200, °C400). The second error is that the picture in the NC group (°C200, green border in **Figure 2G**-Original Version) should be the staining results of the MC group (°C200).The third error is that the HE staining results of the NC group (°C200 in **Figure 2G**-Original Version) are missing.

The corrected pathology pictures “H&E stains of serial sections of colons” of **Figure 2G** in the correct version and its caption “CMF treatment reduced the incidence of CRA in mice. C57BL/6 mice were subjected to an AOM-based CRC induction protocol using three cycles of 2.5% DSS in drinking water. **(A)** Diagram shows the experimental course of AOM/DSS mouse model. **(B)** Body weights of AOM/DSS group and AOM/DSS + CMF group (1, 3, 4, 5, 6). **(C)** Histogram showing the size distribution of tumors. **(D–F)** Tumor sizes in different parts determined by Spot software for microscopic tumors or a caliper for macroscopic tumors. Average tumor size ± S.D. is shown; **(G)** H&E stains of serial sections of colons. *P < 0.05; **P < 0.01. Data are presented as mean ± SD of mice in each group” appear below.

**Figure 2:**
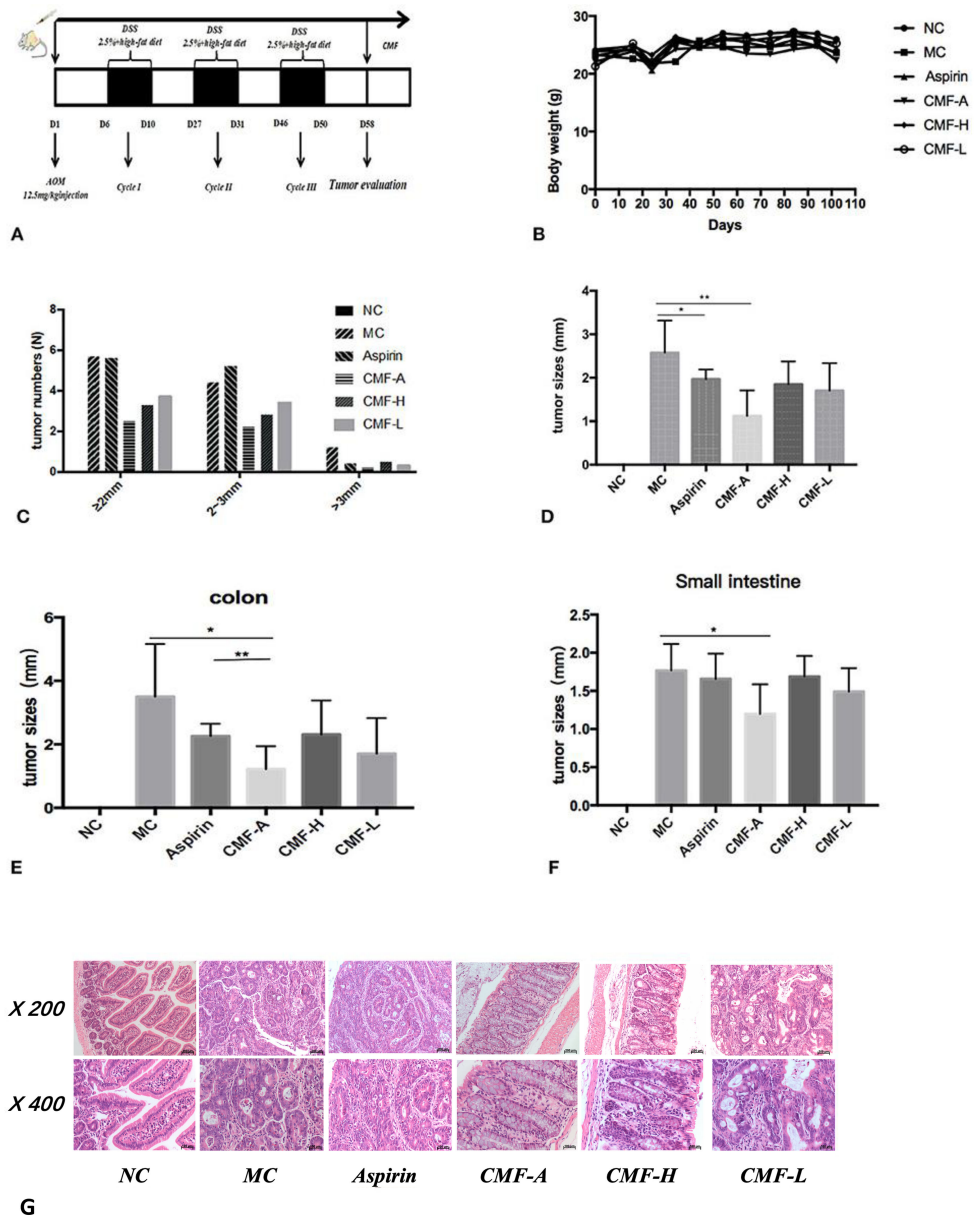
CMF treatment reduced the incidence of CRA in mice. C57BL/6 mice were subjected to an AOM-based CRC induction protocol using three cycles of 2.5% DSS in drinking water. **(A)** Diagram shows the experimental course of AOM/DSS mouse model. **(B)** Body weights of AOM/DSS group and AOM/DSS + CMF group (1, 3, 4, 5, 6). **(C)** Histogram showing the size distribution of tumors. **(D–F)** Tumor sizes in different parts determined by Spot software for microscopic tumors or a caliper for macroscopic tumors. Average tumor size ± S.D. is shown; **(G)** H&E stains of serial sections of colons. *P < 0.05; **P < 0.01. Data are presented as mean ± SD of mice in each group

The authors apologize for this error and state that this does not change the scientific conclusions of the article in any way. The original article has been updated.

